# The journey of SARS-CoV-2 in human hosts: a review of immune responses, immunosuppression, and their consequences

**DOI:** 10.1080/21505594.2021.1929800

**Published:** 2021-07-12

**Authors:** Amal F. Alshammary, Abdulrahman M. Al-Sulaiman

**Affiliations:** a Department of Clinical Laboratory Sciences, College of Applied Medical Sciences, King Saud University, Riyadh, Saudi Arabia; b Department of Medical and Molecular Virology, Prince Sultan Military Medical City, Riyadh, Saudi Arabia

**Keywords:** COVID-19, SARS-CoV-2, coronavirus, severe acute respiratory syndrome coronavirus 2, immunosuppression, leukocytopenia, lymphocytopenia, cytokine storm

## Abstract

Coronavirus disease 2019 (COVID-19) is a highly infectious viral disease caused by severe acute respiratory syndrome coronavirus 2 (SARS-CoV-2). Laboratory findings from a significant number of patients with COVID-19 indicate the occurrence of leukocytopenia, specifically lymphocytopenia. Moreover, infected patients can experience contrasting outcomes depending on lymphocytopenia status. Patients with resolved lymphocytopenia are more likely to recover, whereas critically ill patients with signs of unresolved lymphocytopenia develop severe complications, sometimes culminating in death. Why immunodepression manifests in patients with COVID-19 remains unclear. Therefore, the evaluation of clinical symptoms and laboratory findings from infected patients is critical for understanding the disease course and its consequences. In this review, we take a logical approach to unravel the reasons for immunodepression in patients with COVID-19. Following the footprints of the virus within host tissues, from entry to exit, we extrapolate the mechanisms underlying the phenomenon of immunodepression.

## Introduction

Coronavirus disease 2019 (COVID-19) is a highly infectious viral disease caused by severe acute respiratory syndrome coronavirus 2 (SARS-CoV-2) [1]. COVID-19 was first identified in late December 2019, when a cluster of patients were diagnosed with pneumonia of unknown cause. These patients were linked epidemiologically to the seafood and wet animal wholesale market in Wuhan, Hubei Province, China [2]. Soon after, the disease spread globally, leading to declaration of the COVID-19 pandemic by the World Health Organization [3]. As of April 2021, the disease has spread to 192 countries, with more than 136 million confirmed cases and over two million deaths [4], thus becoming the first pandemic of this century and a critical concern worldwide.

Laboratory findings from a significant number of patients with COVID-19 indicate the presence of leukocytopenia, specifically lymphocytopenia [5,6]. Leukocytopenia is a marked decrease in all white blood cells (WBCs), including myeloid and lymphoid-derived WBCs (i.e., dendritic cells (DCs), macrophages, neutrophils, eosinophils, basophils, natural killer (NK) cells, B cells, and T cells). Lymphocytopenia is defined as a significant decrease of only lymphoid-derived WBCs (i.e., NK, T, and B cells). Both leukocytopenia and lymphocytopenia result in the clinical state of immunodepression/immunosuppression. Moreover, infected patients have been shown to experience contrasting outcomes depending on lymphocytopenia status. Patients with resolved lymphocytopenia are more likely to recover, whereas critically ill patients with signs of unresolved lymphocytopenia develop severe complications, sometimes culminating in death [5–7].

Lymphocytopenia is clearly not an ideal setting in which to fight a viral infection; however, the occurrence of this clinical picture in patients with COVID-19 is unsurprising, as it has been observed in other severe viral infections, including Avian-flu, Swine-flu, SARS, and MERS [8–13]. Nevertheless, lymphocytopenia in the face of viral infection contradicts the physiological function of immune cells, whose sole purpose is to rid the body of foreign entities. Thus, either the invading microbe (in this case, SARS-CoV-2) induces this depression to favor its own survival inside host cells, or there is a failure of the host immune system to fight the disease. Based on this assumption, it is critically important to investigate the underlying pathophysiological mechanisms in patients with COVID-19. The lymphocytopenia observed in these patients is a critical sign of a disrupted immune response, which is alarming, as immune irregularities render patients prone to disease. Moreover, it is not clear why lymphocytopenia manifests in COVID-19 patients. Therefore, the evaluation of clinical symptoms and laboratory findings from infected patients is critical for understanding the disease course and its consequences.

This review takes a logical approach to unravel the causes of immunodepression, specifically lymphocytopenia, in COVID-19 patients by following the footprints of the virus within host tissues from entry to exit and collating information about the mechanisms underlying this phenomenon. Given the relatively short period of time since the emergence of SARS-CoV-2, conclusive data on SARS-CoV-2 pathophysiology inside host tissues are scarce. Therefore, where relevant, host immunological events common in response to coronavirus family members are summarized and related to SARS-CoV-2 or coronavirus family members more generally.

## Clinical manifestations and transmission in COVID-19

Infection with SARS-CoV-2 does not automatically result in disease, and infected subjects can be classified into four main groups according to clinical presentation. The first group are asymptomatic, with no signs or symptoms observed clinically; however, anosmia (loss of smell) and dysgeusia (loss of taste) are common among patients positive for the virus even in the absence of other symptoms [14–16]. The second group present with flu-like symptoms, including fever, fatigue, sputum production, sore throat, and cough [5,6]. The third group present with further symptoms, ranging from mild to severe, including headache, unproductive cough, persistent chest tightness, and difficulty breathing [2,5]. Additionally, a few cases have reported nausea, vomiting, and diarrhea, although this is uncommon [7,17]. Finally, as the disease progresses, the fourth group present with severe life-threatening complications, including pneumonia, acute lung injury (ALI), acute respiratory distress syndrome (ARDS), multiple organ failure, septic shock, and sometimes, death [18,19]. Asymptomatic subjects are considered possible carriers and may or may not spread the infection, whereas symptomatic patients are highly contagious [20–22]. Reports vary greatly with regard to the incubation period from infection to disease onset, and have included ranges as restricted as 5–6 days and as extended as 2–14 days [23,24]. Moreover, preliminary reports estimate the basic reproduction number (R_0_) of the virus is between 1.4 and 3.8, indicating pandemic potential and the possibility of sustained infections within communities [25–28].

Although it is early to calculate infection and fatality frequencies among infected populations, preliminary reports from several affected countries demonstrate that the majority of severe cases and fatalities are among older adults and patients with underlying illnesses [29,30]. Moreover, although it has been reported that fatalities occur among young adults and not in children, reports of children dying from the disease have begun to emerge [31–33]. Furthermore, infected male patients are more vulnerable to the disease than female patients, as the majority of patients who develop severe complications or death are male [34]. In addition, earlier reports showed that pregnant women with confirmed COVID-19 developed symptoms similar to non-pregnant COVID-19 patients [35–38]. However, recent studies reported that COVID-19 pregnant women are at high risk of developing severe complications compared to non-pregnant COVID-19 patients [39,40]. Further, pregnant women with confirmed COVID-19 in their third trimester did not show signs of intrauterine vertical transmission to their fetus, thus reported to be at high risk of preterm delivery due to fetal distress [35,36,41].

## SARS-CoV-2 genome organization and expression

**Figure 1.**
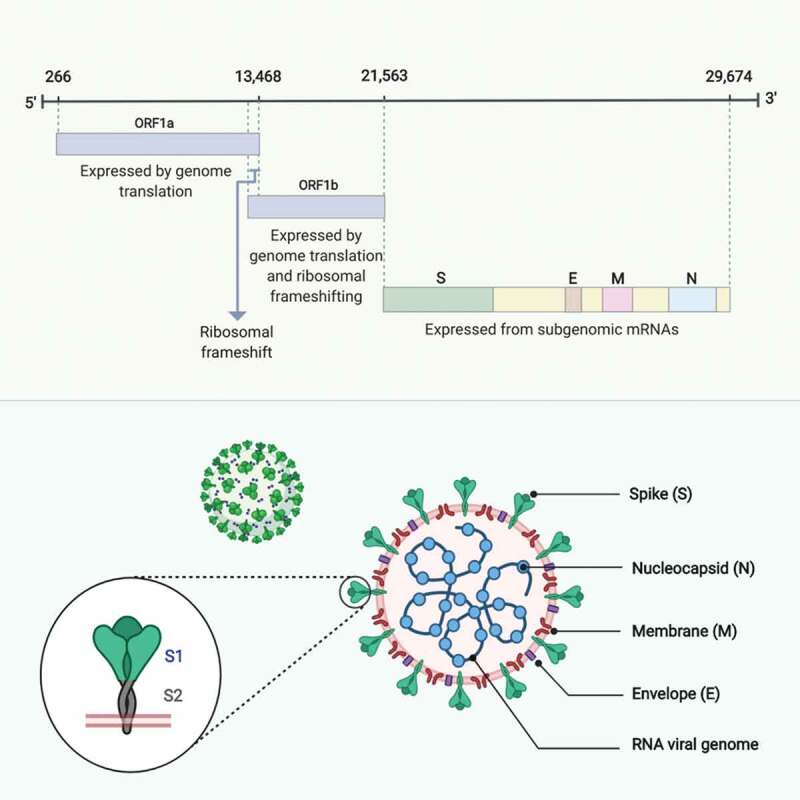
**SARS-CoV-2 genome organization and structural proteins configuration. (A)** SARS-CoV-2 contain a non-segmented positive sense single-stranded RNA (+ssRNA) genome of about 30 kb, organized as follows: 5ʹ–untranslated region (UTR), replicase open reading frame 1ab (ORF1a/ORF1b), spike glycoprotein (S), envelope protein (E), membrane protein (M), nucleocapsid protein (N), non-structural open reading frames (NS-ORFs), 3ʹ–UTR. **(B)** Configuration of the SARS-CoV-2 four main structural proteins: the spike glycoprotein (S), comprised of two subunits, S1 and S2, expressed on the surface of the virion as a club-shaped trimer; the envelope protein (E), a transmembrane protein present in small quantities within the virion; the membrane protein (M), a transmembrane dimer that serves as a connecter binding the virion envelope to the helical nucleocapsid and giving the virion its spherical shape; and the nucleocapsid protein (N), the sole protein constituent of the nucleocapsid capable of binding to the viral genome. Adapted from [49,50]. Made with BioRender [65]

SARS-CoV-2 belongs to the β-coronaviruses, which are known to infect mammals [1]. They contain a non-segmented positive sense single-stranded RNA (+ssRNA) genome of about 30 kb, organized in a typical coronavirus genome pattern as follows: 5ʹ–untranslated region (UTR), replicase open reading frame 1ab (ORF1a/ORF1b), spike glycoprotein (S), envelope protein (E), membrane protein (M), nucleocapsid protein (N), non-structural open reading frames (NS-ORFs), 3ʹ–UTR [1,42,43] (Figure 1A). Two-thirds of the viral RNA (around 20 kb) is occupied by replicase genes and encodes non-structural proteins (NSPs), while the remainder (approximately 10 kb) encodes structural and accessory proteins [44,45].

Coronaviruses have a unique genome configuration as their +ssRNA can act as a messenger RNA (mRNA) from which coding sequences are directly translated into proteins upon reaching the cell translation machinery, namely ribosomes [43,46]. This is possible because the viral genomes contain sequences similar to those of cellular mRNAs with a 5ʹ-cap (leader) and 3ʹ-poly adenylated (A) tail (body) which creates a mature RNA template that can undergo translation. This process usually initiates at the 5ʹ end, which restricts translation to the first ORF; however, coronaviruses have a polycistronic genome configuration. This allows initiation of translation of the first ORF, encoding the replicase polyproteins, which have replicative functions. These proteins can generate nested sub-genomic mRNAs, encoding structural and accessory proteins located downstream of the replicase ORF [47]. This structure allows the transcription machinery to translate both the first viral ORFs and multiple downstream ORFs [48], since each newly created mRNA comprises a 5ʹ-cap/3ʹ-poly (A) tail, which are joined during the discontinuous-extension phase of negative RNA strand synthesis. Virus structural and accessory proteins are eventually translated from these generated sequences [49].

Overall, translation of replicase ORFs, including ORF1a and ORF1b, results in the synthesis of two large replicase polypeptides, pp1a and pp1b, that undergo proteolytic cleavage. This is mediated by internal proteases encoded by ORF-1a NSPs, which process the two replicase polypeptides to yield 15 or 16 active proteases [50]. Consequently, replicase polypeptides initiate the generation of nested RNAs encoding the structural-ORFs, which give rise to the four main structural proteins: S, E, M, and N [51,52]. The S glycoprotein mediates attachment and virus entry into host cells, and is comprised of two subunits: S1, which determines virus-to-host cellular tropism, and S2, which mediates virus-to-cell membrane fusion [53–55]. Both subunits are expressed on the surface of the virion as a club-like shaped trimer, giving the virus the appearance of a solar crown, hence the name, crown-like or corona [53]. The M protein is expressed as a transmembrane dimer and is thought to give the virion its spherical shape [43]. Moreover, M protein is the most abundant envelope protein and may serve as a connecter, binding the virion envelope to the helical nucleocapsid [56]. Similarly, E protein is thought to be a transmembrane protein, and although present in small quantities, appears to play a key role in promoting viral pathogenesis [57–59]. In contrast, the N protein is the sole protein constituent of the nucleocapsid and is capable of binding to the viral genome *in-vitro* [60–62] (Figure 1B). Finally, the translation of accessory protein ORFs generates proteins considered non-essential, which nevertheless promote virus pathogenicity and modulate host innate immunity [63,64].

## The SARS-CoV-2 life cycle

Specific details regarding the SARS-CoV-2 life cycle inside host cells have yet to be determined; however, knowledge of other coronavirus family members, including severe acute respiratory syndrome coronavirus (SARS-CoV) and Middle East respiratory-related coronavirus (MERS-CoV), may provide clues as to the life cycle of SARS-CoV-2 [66]. For these viruses, the life cycle begins upon host entry via respiratory droplets, where they target respiratory epithelial cells, especially alveolar epithelial cells of the lung. These cells express high levels of the receptor needed for entry of SARS-CoV-2, angiotensin-converting enzyme 2 (ACE2), which is the same receptor used by SARS-CoV to infect humans [67–69]. This receptor was quickly identified based on the fact that SARS-CoV and SARS-CoV-2 share 79% similarity [70–72]. ACE2 is found mostly in the lower respiratory tract, where SARS-CoV-2 causes the most damage [73]. Further, SARS-CoV-2 has been detected in bronchoalveolar lavage fluid (BALF) isolated from a patient with COVID-19 [70].

SARS-CoV and MERS-CoV follow similar entry routes after cellular attachment; however, MERS-CoV binds to the dipeptidyl peptidase 4 (DPP4) receptor on host cells. Regardless of the entry receptor, these viruses target respiratory epithelial cells [74–77]. Entry into host epithelial cells is initiated once the virus particle is engulfed with its envelope intact. Once inside the endosomal vesicle, induced lysosomal proteases cleave the viral S glycoprotein, mediating fusion of the viral envelope with the host endosomal membrane [78,79]. Subsequently, the naked virion is released into the cytoplasm where nucleocapsid proteins disassociate, liberating the viral +ssRNA [80,81]. Once the +ssRNA is released into the cytoplasm, it moves to host ribosomes where the genome is decoded, proteins are synthesized, and infectious progeny are produced [82].

Coronavirus replication and assembly is associated with both the rough endoplasmic reticulum (ER) and Golgi complex, as several studies have revealed that virion particle formation occurs in the intermediate compartment of smooth-walled tubulovesicular membranes between the ER and Golgi complex (ERGIC) [83–89]. Following translation, the viral structural proteins, S, E and M, are inserted into the ER and eventually transported into the secretory pathway of the ERGIC, forming empty, assembled virus-like particles (VLPs) [90–92]. Subsequently, the translated N protein polymerizes around the newly synthesized viral genome to form the nucleocapsid [93]. Finally, the N-protein-encapsidated full-length viral genome buds into the ERGIC complex, which contains associated envelope and structural proteins, to form a mature virion [94,95].

**Figure 2.**
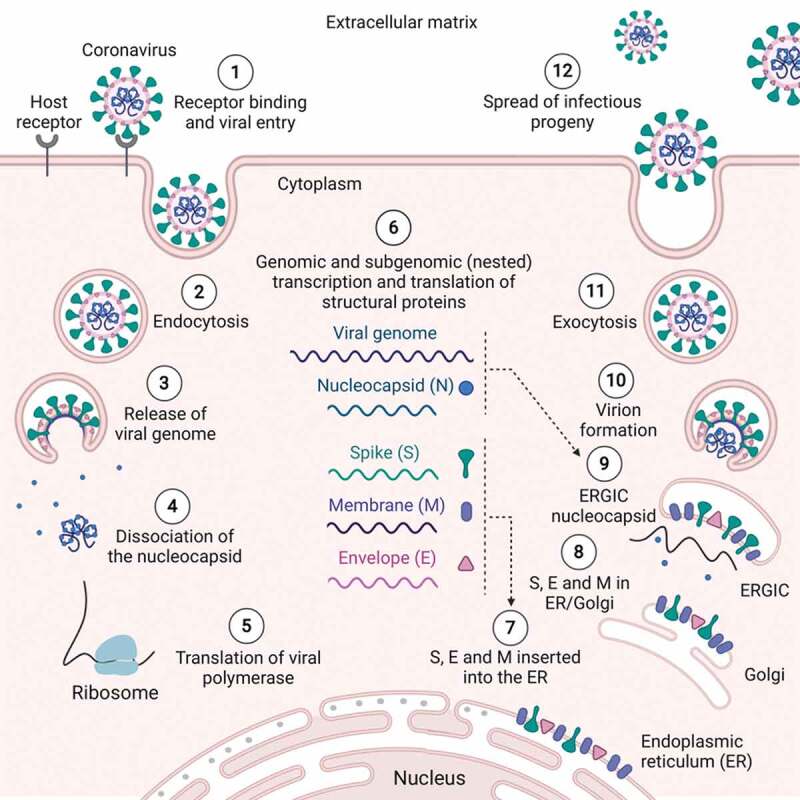
**The SARS-CoV-2 life cycle** [1]. The S glycoprotein on the surface of SARS-CoV-2 facilitates its entry into respiratory epithelial cells through binding to the ACE2 receptor [2]. Entry is initiated via endocytosis once the virus particle is engulfed with its envelope intact [3]. Inside the endosomal vesicle, induced lysosomal proteases start to cleave viral S glycoproteins, mediating fusion of the viral envelope with the host endosomal membrane [4]. The naked virion is released into the cytoplasm, where the nucleocapsid proteins start to disassociate, liberating the viral +ssRNA [5]. Released +ssRNA in the cytoplasm moves to host ribosomes where the genome is decoded. The polycistronic genome configuration allows the translation of the first ORF, which encodes the replicase polyprotein (polymerase) [6]. Replicase then generates the viral genome and nested sub-genomic mRNAs, which encode the four main structural proteins; spike (S), envelope (E), membrane (M), and nucleocapsid (N) [7]. Following translation, the viral structural proteins, S, E, and M, are inserted into the ER [8] and eventually transported into the secretory pathway of the ER and golgi complex (ERGIC), forming empty, assembled virus-like particles (VLPs) [9]. Translated N proteins subsequently polymerize around the newly synthesized viral genome to form the nucleocapsid, which buds into the ERGIC complex containing associated envelope and structural proteins to form a mature virion [10]. The N-protein-encapsidated full-length viral genome forms new virions [11]. Assembled virions exit via exocytosis [12], and newly formed virus particles spread to infect adjacent tissues. Adapted from [44]. Made with bioRender [65]

Assembled virions exit the cell in the same way they enter, within an endosomal vesicle, and thus their outbound route is via exocytosis [96] (Figure 2). However, mature virus particles do not exit separately, as multiple virions are engulfed inside a giant endosomal vesicle from which they exit together as a group [43]. This deceptive egress allows the virus to spread quickly before being detected by resident and circulating immune cells near the infected cells. Electron microscopy (EM) visualization of SARS-CoV-2-infected epithelial cells isolated from a patient with COVID-19 showed giant vesicles containing multiple virions present in the cytoplasm, whereas single virions were observed extracellularly. Thus, this confirms this method of egress, or budding, following infection [2].

## Cell injury following SARS-CoV-2 infection

SARS-CoV-2 infection causes severe damage to epithelial cells, triggering cell death. As the virus continues its invasion into neighboring cells, the epithelial lining becomes excessively inflamed and damaged, eventually leading to the development of ARDS [7,97]. It is not yet clear how cell death occurs following SARS-CoV-2 infection; however, lung autopsy reports from patients with SARS-CoV and MERS-CoV revealed epithelial cell necrosis, alveolar fibrosis, and diffuse alveolar damage [98–100]. These histological features suggest that necrotic death, rather than autophagy or apoptosis, occur following infection. This may be partly due to the fact that the virus is shed through transport within a large vesicle that eventually merges with the plasma membrane thus destabilizing its surface. Following infection with coronaviruses, epithelial cells lose their ciliated surfaces. This damage may be associated with the newly formed endosome-plasma membrane structure [100–102]. Indeed, the dry cough associated with severe pneumonia supports this hypothesis, as it is a clinical symptom that results from reduction of or damage to the ciliated surface responsible for the movement of mucous. It should be noted, however, that several studies have demonstrated the induction of apoptosis in tissues from multiple organs following infection with SARS-CoV-2 [103,104]. Thus, we speculate that induced apoptosis is a consequence of a dysregulated immune response, while necrotic cell death is the result of viral replication inside permissive cells. Thus, non-permissive cells may undergo induced apoptosis following intracellular immune recognition of viral components. Moreover, it is also possible that necrotic cell death predominates during the early stages of infection. As the disease progresses and immune dysregulation becomes prominent, apoptosis or autophagy may become the main cause of cellular death. Indeed, it has been previously proposed that cell death can be induced via different mechanisms during infection, although more studies are needed to prove or disprove these hypotheses [105].

While autophagy or apoptosis of permissive epithelial cells are possible during the early stages of viral infections, they are unlikely for two reasons [1]: the extensive damage observed in the lung, including fibrosis, do not usually occur following autophagy or apoptosis [2]; the exaggerated immune reaction and cytokine production at the site of infection indicate necrotic cell death, rather than autophagy or apoptosis, which are considered clean types of cell death not requiring a heightened immune response. This is important, as both the virus replicative cycle and resultant cellular injury define the nature of the activated immune responses. As the virus sheds, host immune cells should recognize either the virus, the injured cell, or both. Consequently, it is critical to understand the nature of the immune reactions exerted in this scenario, as both can result in inflammation that translates to excessive cytokine production, known as a *cytokine storm*. Understanding this concept leads to the logical conclusion that treatment targets must be based on the underlying cause. Thus, whether a cytokine storm arises due to virus recognition or cell death must be determined to identify the cause of immunodepression observed in patients with COVID-19 and to find an effective treatment solution.

## Immune recognition

The human immune system has a dual nature, with innate and adaptive arms. Innate immunity is nonspecific, and recognizes antigens without the need for previous encounters with invading microbes. Innate immune cells include: DCs, macrophages, neutrophils, eosinophils, basophils, and NK cells. In contrast, adaptive immunity is specific and requires antigen presentation through antigen presenting cells (APCs) (primarily DCs), which activate adaptive immune cells (B and T cells) to produce effector progeny that can combat disease in a directed manner. Because SARS-CoV-2 has not previously encountered by humans, the innate immune response to this pathogen is likely of central importance.

Innate immune cells recognize structurally conserved molecules through pattern recognition receptors (PRRs), which can be membrane bound, such as Toll-like receptors and C-type lectin receptors, or cytoplasmic, such as nucleotide oligomerisation domain-like receptors and RIG-I-like receptors [106–108]. Moreover, depending on the nature of the target antigens, innate PRRs recognize and bind two types of structures. If foreign (non-self) antigens are present, PRRs bind to common pathogen structures, known as pathogen-associated molecular patterns (PAMPs) [109,110], whereas PRRs bind to damage or danger associated molecular patterns (DAMPs), also known as alarmins, on non-foreign (self) antigens [111–113]. PAMPs include signature microbial structures, such as surface glycoproteins, cellular and non-cellular structures, and microbial nucleic acid [114]. In contrast, DAMPs include a varying repertoire of host biomolecules that are usually intact or confined within the cell, but are released as a result of cell injury or necrotic cell death. These include degraded extracellular matrix proteins or intracellular molecules (cytosolic and nuclear proteins) [115].

In COVID-19, the virus can be detected in patient samples, leading to the assumption that PPRs must be binding to PAMPs (viral components). Moreover, patient radiographs, CT scans, and laboratory profiles confirm that active cellular damage occurs and thus, PPRs must also be binding to DAMPs (cellular components). Surprisingly, whether binding PAMPs or DAMPs, PRRs activate similar pathways, regardless of the nature of the stimulating antigen [116–119]. This phenomenon is beneficial, as it reflects immune system recognition of the cellular damage mediated by microbial infection. Further, this strategy highlights the sophistication of the human immune system, which helps to restrict both microbial replication and cellular damage from reaching other organs and causing additional complications. Thus, the magnitude of the immune response corresponds to the number of inducing antigens (self and non-self) present at the site of infection [117,120]. Understanding the consequences of PRRs binding to PAMPs and DAMPs will enable further understanding of the clinical picture presented in COVID-19.

### Innate immune responses

PAMP/DAMP-associated molecules at the site of infection trigger a potent innate immune response that corresponds to the extent of tissue damage [121–123]. More virus components and increased cell damaged result in augmented recruitment of innate immune cells to the site of injury, leading in turn to greater cytokine production. This phenomenon can result in a systemic hyper-inflammatory response, known clinically as systemic inflammatory response syndrome (SIRS) [120,123,124]. Several lines of evidence indicate that, following SIRS, a secondary immune response, known as the SIRS-like immune response, is initiated to boost antimicrobial defense mechanisms [125,126]. This strategy is remarkable, given that extensive damage and immune recruitment to the site of injury can leave the body defenseless against resident microbial flora, which will inevitably cause added insult to tissues and organs. Both, SIRS and SIRS-like immune responses result in the clinical phenomenon described as a cytokine storm [126–128]. The immune system senses the level of damage caused by this exaggerated cytokine release, which can overwhelm the physiological system, leading to unintended damage. Consequently, a third immune response is induced to reduce the collateral damage generated by the cytokine storm. This third response manifests as an anti-inflammatory response, known as counterbalancing compensatory anti-inflammatory response syndrome (CARS) [129–131]. Initiation of CARS induces downregulation of immune cells, reflected in the numbers of lymphocytes observed following injury and resulting in a condition referred to as post-traumatic immunosuppression [132,133]. SIRS/SIRS-like responses and CARS are contrasting phenomena, the first results in hyper-inflammatory cytokine production, whereas the latter produces a reduction in inflammatory cytokines. Both syndromes occur simultaneously and correlate with patient status and the extent of injury [132,134]. SIRS is mediated by macrophages, while SIRS-like secondary immune responses are predominantly mediated by both macrophages and neutrophils, and CARS is mediated by T cells [135–139]. Patients can present with any of the following: SIRS, with a pro-inflammatory response; mixed SIRS and CARS with both pro- and anti-inflammatory responses; and finally CARS with an anti-inflammatory response [125,132,135].

This dual immune response is observed in patients with severe COVID-19, as laboratory findings support the co-occurrence of a cytokine storm and immunosuppression. Surprisingly, this clinical/laboratory picture is a mirror image of what happens during severe trauma, as microbial and non-microbial injuries result in sepsis, septic-shock, and multiple organ failure [140,141]. Thus, a rapid review reveals that COVID-19 mimics the pathophysiology observed in response to severe injuries. This observation indicates that what is known about the nature of immune responses and cytokine production in trauma can be applied to COVID-19, which could aid in the pursuit of better solutions to combat this disease.

### Ground-zero cytokines

Upon identification of a cytokine storm following injury, the type of cytokines present and cells responsible for their secretion must be identified. Both immune and nonimmune cells are known to secrete specific sets of cytokines with overlapping functions. Therefore, it is difficult to determine the nature of cytokines produced and which cells are responsible, since many cells are involved in this scenario. However, a logical approach is to follow the causative agent and identify the first cellular responder. This presumed first contact will lead to secretion of cytokines responsible for the recruitment and activation of corresponding cells, which in turn secrete their own cytokines generating a cytokine network. The causative agent and first cellular responder produce what are known as *ground-zero cytokines*, from which all following reactions stem.

The underlying mechanisms occurring within the respiratory system in response to inhaled particles enable reconstruction of the pathway from which the causative agent, in this case SARS-CoV-2, emerges from respiratory droplets reaching the lung. Normally, inhaled particles larger than 10 μm are deposited onto the mucous-coated ciliated-epithelium surface lining the nose, pharynx, trachea, and conducting airways [142]. Such mucous-trapped particles in the lower conducting airways are then expelled through a process known as the mucociliary escalator, whereby the beating movement of ciliated-epithelia transports mucous-trapped particles in an upward motion toward the mouth [142–144]. This process is facilitated by the functions of sneezing, coughing, and swallowing mucous-trapped particles. In contrast, inhaled particles smaller than 5 μm pass through the conducting airways and land on the surface of bronchiolar-respiratory duct junctions or, alternatively, are deposited onto the surface of alveoli [142]. At this site both resident alveolar macrophages, located adjacent to lung alveoli, and pulmonary DCs, located beneath the alveolar basement membrane, provide protection from incoming inhaled particles [145].

Due to their size, SARS-CoV-2 particles most likely enter in respiratory droplets and land on the surface of lung alveoli. ACE2 receptor expression and alveoli damage following infection support this theory [2,72]. From there, some virus particles will come in contact with resident alveolar macrophages, some will be sampled by lung-resident DCs, and others will escape and go on to infect alveolar epithelial cells, which may recognize the virus after it begins its replicative cycle in the cytoplasm [146–148]. In contrast, alveolar macrophages, given their location adjacent to the alveolar epithelium and the fact that their numbers exceed those of lung-resident DCs, recognize virus particles on contact. This reconstructed pathway suggests that resident alveolar macrophages are likely the first responders, followed by alveolar epithelial cells and lung-resident DCs. Based on this assumption, alveolar macrophages would be responsible for production of the ground-zero cytokines referred to above. The next question to explore is the nature of these cells and the mechanisms underlying their cytokine secretion, as well as the cells they communicate with and their corresponding cytokine responses. Decoding cellular communications in this setting will aid efforts to reveal the nature of the cytokine storm produced following SARS-CoV-2 infection.

### Cellular recruitment and cytokine production

Resident alveolar macrophages are professional phagocytes, constituting more than 90% of total hematopoietic cells present in bronchoalveolar lavage fluid [149–152]. Their main function is to protect the airway from foreign microbial and non-microbial antigens, as well as to clear cellular debris; however, they are also responsible for post-infection remodeling of the lung parenchyma [153–159]. These macrophages maintain lung homeostasis by preventing unnecessary immune responses against harmless inhaled particles, which could cause unnecessary lung injury and interfere with alveolar-gas exchange [160–162]. Resident alveolar macrophages maintain this dampened immune characteristic of the lung by producing anti-inflammatory cytokines necessary to block unwanted alveolar inflammation [163–165]. Several cytokines produced by resident alveolar macrophages can supress alveolar inflammation, including transforming growth factor β (TGFβ) [166,167]. Additionally, secretion of interleukin 10 (IL-10) and prostaglandin E_2_ supresses adaptive immunity through blocking the activation of both DCs (communicators with adaptive cells) and T cells [160,162]. Similarly, alveolar epithelial cells produce IL-10 and supress the activation of alveolar macrophages [168]. Moreover, lung-specific surfactant proteins A and D (SP-A/SP-D) have key roles in maintaining the suppressive phenotype of resident alveolar macrophages through binding to calreticulin and CD91 receptors on the surface of resident alveolar macrophages [169,170]. It should be noted that during infections SP-A and SP-D bind to PAMPs removing their suppressive function on resident alveolar macrophages. As a consequence, CD91 on the surface of these macrophages is free and instead bind to PAMPs [169]. CD91/PAMP signaling alters the phenotype of resident alveolar macrophages, which begin to secrete pro-inflammatory cytokines with direct effects on the alveolar epithelium and serve as inflammatory signals for the recruitment of circulating monocytes and polymorphonuclear cells.

IL-1β and tumor necrosis factor α (TNFα) are the first cytokines produced by resident alveolar macrophages in response to microbial stimulants [142]. Due to their location, the first responders to these cytokines are alveolar epithelial cells, which upregulate the expression of chemokine ligand 2 (CCL2), thereby attracting circulating monocytes and neutrophils expressing chemokine receptor 2 (CCR2) to the site of infection [171–174]. Additionally, several chemokines are upregulated in alveolar epithelial cells following lung infections, including CCL3, RANTES (CCL5), IL-8/CXCL8, and interferon-γ-inducible protein (IP10/CXCL10) [174]. Moreover, studies on animal models susceptible to SARS-CoV infection revealed that alveolar epithelial cells upregulate the expression of potent monocyte and neutrophil recruitment molecules, such as the adhesion molecules P-selectin, vascular cell adhesion protein 1 (VCAM-1), and DC-specific intracellular adhesion molecule-3-grabbing non-integrin (DC-SIGN) [174,175]. During viral infections (other than coronaviruses), virus-infected epithelial cells secrete interferon β (IFNβ) causing neighboring cells to produce anti-viral proteins that inhibit viral replication in non-infected cells. Of note, SARS-CoV induces a dysregulated immune response by blocking the production of several pro-inflammatory cytokines and chemokines including, IFNα, IFNβ, IFNγ, TNF, CCL5 and CXCL10 [174–178].

The next immune responders are lung-resident DCs, which are situated at the basolateral side of the epithelium and extend their projections (dendrites) into the lumen of alveoli and conducting airways to sample antigens without disrupting the intact epithelial barrier layer [179–182]. Once antigens are captured, lung-resident DCs migrate to local draining lymph nodes to present processed antigens to T cells [183,184]. Normally, lung-resident DCs express E-cadherin-binding integrin (αEβ7), and CCR6 [185–187]. Following viral infection, these DCs immediately respond to TNFα and CCL20, inducing their dispatch to local lymph nodes for antigen presentation [188,189]. However, DC activation does not trigger local inflammation, as T cell activation following bacterial challenge is minimal [162,179,190]. Moreover, several studies have shown that T cells extracted from mucosal lymph nodes exhibit Th2 polarity upon activation [191,192]. Studies on animal models indicate that the DC population is heterogenous. However, two classical subtypes can be identified by their cytokine production and ability to stimulate specific T cell subsets, IL-12 DCs, which stimulate proliferation of Th1 cells, and IL-10 DCs, which stimulate Th2 cell proliferation [193]. Collectively, these findings confirm the regulatory role of lung-resident DCs in preventing local inflammation during lung infections.

Endothelial cells are another key local responder during lung infections. These cells line blood vessels beneath the basement membrane of lung alveoli providing the ideal interface for gas exchange. However, inflammatory mediators released by alveolar epithelial cells and alveolar macrophages promote vasodilation and increase permeability to permit leukocyte influx into infected areas. Moreover, released cytokines travel through blood vessels and subsequently stimulate the recruitment of circulating leukocytes. In addition, vasodilation causes fluid to escape into the interstitial space beneath lung alveoli resulting in edema and alveolar shrinkage, which can progress to development of pneumonia, ALI, and ARDS [194,195]. Studies on influenza viral infection of the lung revealed induction of pro-inflammatory cytokine expression by endothelial cells, including IL-6, IL-8, TNFα and TNFβ [196]. Collectively, endothelial cytokines may contribute to the cytokine storm observed following lung infection and consequent localized edema. This can result in systemic infiltration of fluids affecting multiple organs, and not just the lung.

Regarding cellular responders from outside the lung, neutrophils arrive to the injury site within minutes and, under normal circumstances, are the first to die, within 24 hours of their dispatch from the bone marrow [197–199]. Neutrophils participate in microbial clearance through their production of hydrogen peroxide (H_2_O_2_), superoxide anion, microbiocidal proteins, and extracellular traps (NETs). However, following severe trauma or viral infection, neutrophils can exhibit a prolonged lifespan through their ability to resist intracellular or extracellular apoptotic signals under such circumstances [199–203]. Consequently, an increase in circulating neutrophils, referred to as neutrophilia, is a common outcome in many viral infections [204]. Further, neutrophils with prolonged lifespans exhibit heightened aggression, causing indirect damage within injured tissues [205–210].

The next cells to contribute are circulating monocytes, which differentiate into macrophages upon reaching tissues. The demands encountered by monocytes at target locations dictate their differentiation into specific macrophage subtypes, each with unique characteristics and cytokine profiles [211]. Subtype I macrophages are responsible for clearing cellular debris and dead neutrophils, and secrete anti-inflammatory cytokines, such as tumor growth factor β (TGFβ) and IL-10 [212–214]. In contrast, subtype II macrophages recognize DAMPs and secrete pro-inflammatory cytokines, such as IL-1β, IL-6, IL-8/CXCL8 and TNFα, in addition to inducible nitric oxide synthases [215–221]. Moreover, subtype II macrophages produce several chemokines, including CCL2, CCL7 and CCL12, resulting in further accumulation of recruited inflammatory cells [222,223]. Based on their cytokine production, subtype I macrophages are mainly responsible for the repression of adaptive immune cells, whereas subtype II macrophages are responsible for the cytokine storm observed following lung infections [224,225].

**Figure 3.**
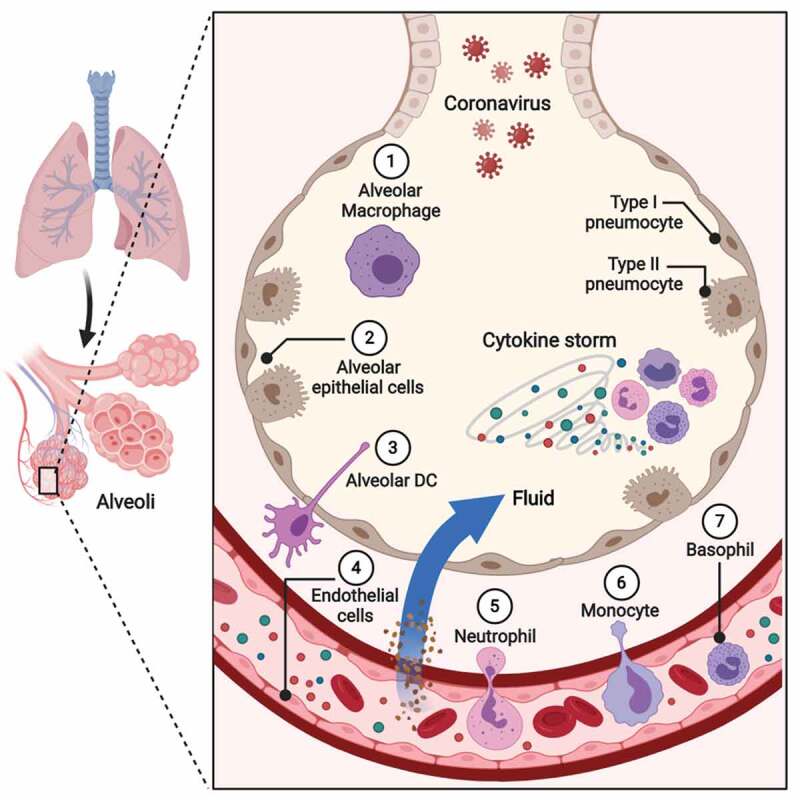
**Innate immune responses in lung infections**. Coronavirus particles causing COVID-19 most likely enter the body via respiratory droplets and land on the surface of lung alveoli. From there, some virus particles will come in contact with [1] resident alveolar macrophages followed by [2] alveolar epithelial cells. The next immune responders are [3] lung-resident dendritic cells situated at the basolateral side of the epithelium with projections (dendrites) extended into the lumen of alveoli and conducting airways to sample antigens without disrupting the intact epithelial barrier layer. Another key local responder during lung infection is [4] endothelial cells. These cells line blood vessels beneath the basement membrane of lung alveoli. However, inflammatory mediators released by alveolar epithelial cells and alveolar macrophages promote vasodilation and increase permeability to permit leukocyte influx into infected areas. Moreover, released cytokines travel through blood vessels and subsequently stimulate the recruitment of circulating leukocytes. In addition, vasodilation causes fluid to escape into the interstitial space beneath lung alveoli, resulting in edema and alveolar shrinkage. Regarding cellular responders from outside the lung [5], neutrophils arrive to the injury site within minutes. The next cells to contribute are [6] circulating monocytes, which can differentiate into macrophages on reaching tissues. Another key player in this cellular constellation is [7] basophils (mast cells within tissues), readily available to respond to invading microbes, as they are strategically located near blood vessels, regional lymph nodes and nerve endings, allowing them to modulate host responses to invading pathogens. Made with BioRender [65]

Mast cells are another key player in this cellular constellation. Further, the role of mast cells in lung immunopathology following viral infections is often underestimated. Readily available to respond to invading microbes, mast cells are strategically located near blood vessels, regional lymph nodes, and nerve endings, allowing them to modulate host responses to invading pathogens [226,227]. Mast cells contain powerful granular mediators that are immediately released upon activation, including TNFα, β-hexosaminidase, amines, histamine, serotonin, antimicrobial peptides and proteases (tryptases and chymases) [228–231]. In addition, mast cells release preformed granules within a few minutes of an injury containing factors such as prostaglandins and leukotrienes. Preformed cytokines and growth factors, including IL-4, IL-5, IL-6, IL-13, IL-17, TNFα and vascular endothelial growth factor (VEGF), are released by mast cells a few hours later [231–233]. Cytokines, chemokines, and chemical mediators released by mast cells following infection lead to increased epithelial and endothelial cell permeability, which in turn results in increased cellular recruitment and infiltration (Figure 3).

Although the cytokine storm produced following lung injury or infection is responsible for the immunopathology observed in patients with COVID-19, immune cell recruitment to injured tissues mainly involves innate cells, such as neutrophils, monocytes and mast cells, rather than adaptive immune cells. Thus, the purpose of the cytokine storm is thought to be the recruitment of myeloid, rather than lymphoid, cells. Nevertheless, lymphoid cell fate during viral lung infection is crucial, since the presence of certain lymphocyte populations can predict the severity and outcome of COVID-19.

### Lymphocyte subsets

A reduction in lymphocytes is associated with severe injuries and unresolved infections [234–243]. The initiation of a cytokine storm following infection clearly contributes to this reduction, as the storm serves to recruit myeloid, rather than lymphoid, cells [244,245]. However, it should be noted that DCs, which are of myeloid origin, are also reduced [246,247]. This phenomenon can be explained through understanding of the basic physiological function of these cells. DCs are professional phagocytic cells, which primarily ingest foreign particles and later present them to T helper (Th) cells, thereby serving as the main initiators of adaptive immune responses. *In vitro* studies have shown that naïve T cells are only stimulated by DCs, which may explain why a reduction in DC counts prevents activation of adaptive immune responses [248]. Moreover, several studies have demonstrated that apoptosis is induced following viral infection resulting in a massive depletion of follicular DCs in addition to T cells (CD4 and CD8), B cells, and NK cells [246,249,250]. In addition, circulating CD4 and CD8 T cells exhibit increased expression of programmed cell death protein 1 (PD-1), which inhibits their activation and cytotoxic functions [251–254].

Although T cell subsets are reduced following a cytokine storm, this reduction is selective and depends on the T cell subset. Several studies indicate that CD4^+^CD25^+^Foxp3^+^ T regulatory (Treg) cells are increased following viral sepsis and contribute to the depressed immune environment through their increased production of IL-10 [242,255–257]. Unlike CD4 and CD8 T cell subsets, Tregs are resistant to sepsis-induced apoptosis and cause further insult to CD4 T cells, inducing their elimination or apoptosis through activation of the transforming growth factor β1 (TGFβ1) signaling pathway [237,258–260]. Moreover, increased Treg numbers indicate an inferior condition in septic shock patients and are associated with poor outcomes [256,257]. Similarly, CD4 T cells biased toward Th2 have been linked to a fatal outcome following viral sepsis [261–263]. This was linked to Treg secretion of IL-10, which promotes Th2 polarization of immune cells [262,264]. *In vitro* studies revealed that the major source of IL-10 secretion is Th1/2 and Th17 cells [265,266], which blocks CD4 T cell activation by inhibiting their production of pro-inflammatory cytokines, including IL-2, IL-5, TNFα, IFNγ and GM-CSF [267]. Indeed, IL-10 production by Th cell subsets is thought to regulate immune responses and provide protection from immunopathology during infection, while simultaneously suppressing activation of adaptive immune responses, manifesting clinically as the immunosuppression observed in unresolved infections.

## Lymphocytopenia in COVID-19

A reduction of peripheral lymphocyte counts, including CD4+ and CD8 + T cells, B cells and NK cells, has been observed in COVID-19 patients [268–272]. Further, SARS-CoV-2 has been shown to induce a relative loss of lymphoid cells coupled with myeloid cell expansion in COVID-19 patients [273]. This reduction has been linked to disease severity and survival, as severe and fatal cases show a significant reduction compared to mild or recovered COVID-19 cases [274,275]. Thus, lymphocytopenia is more evident in the elderly and patients with underlying conditions such as hypertension, heart disease, diabetes, chronic obstructive pulmonary disease (COPD) and cancer [29,276,277]. Interestingly, lymphocytopenia is rarely detected in children, with less than 10% of SARS-CoV-2 infected children presenting with lymphocytopenia; however, when lymphocytopenia is detected in children it is usually found to be associated with complicated or severe cases [278–282].

The phenotypic and functional characterization of T cell subsets clearly indicates a defective cellular response in COVID-19 patients. T cells play an essential role in cellular immune regulation and viral clearance. A retrospective study conducted by Diao et al. indicated a 70% decrease in the levels of CD4+ and CD8 + T cells within non-ICU COVID-19 patients, and this reduction was more evident in ICU patients (reaching up to 95%) [283]. The fact that T cell populations, including CD4+ and CD8 + T cells, are reduced in COVID-19 patients suggests impaired or dysregulated cellular immunity in these patients and may be explained by several mechanisms. A study by Notz et al. reported a decrease in naïve Th cells in severe COVID-19 cases [284], which may indicate an impairment of T cell proliferation, maturation, and activation [285]. Another study performed by Rendeiro et al. showed that in COVID-19 patients T cells display overexpression of exhaustion and dysfunction markers such as the v-domain Ig suppressor of T cell activation (VISTA), T cell immunoglobulin and mucin-domain containing-3 (TIM-3), lymphocyte activation gene 3 (LAG-3), T cell immunosuppressor with Ig and ITIM domains (TIGIT), and PD-1 [273,286]. In line with this finding, Chen et al. and Sadeghi et al. reported increased levels of the pro-inflammatory Th17 cells, a major source of IL-10 production [272,287]. Thus, IL-10 secretion may induce T cell exhaustion as animal models of chronic infection have been shown to reverse T cell exhaustion following IL-10 blockade [288,289]. Furthermore, the accumulation of overactivated T cells in the lung during disease may cause reduced T cell counts in the peripheral blood. Wang et al. reported increased expression of the activation marker HLA-DR and the co-stimulatory molecule CD28 in severe COVID-19 patients [290,291]. Consistent with this finding, overactivated T cells have been found to predominate leukocyte infiltration in the lung following SARS-CoV-2 infection, which may reduce circulatory T cells in the periphery. At the same time this would promote T cell exhaustion, as it has been documented during chronic inflammation that continuous recruitment and stimulation of T cells can result in T cell exhaustion [292,293].

However, conflicting findings have been reported regarding Treg counts in COVID-19 patients. Chen et al. reported increased levels of Treg-enriched cells in COVID-19 patients [272]. The same study reported higher CD25 and lower CD127 expression in the Treg population, indicating enhanced functions of Tregs in suppressing the systemic inflammation observed in these patients. On the other hand, Flament et al. found a significant reduction in Tregs among SARS-CoV-2 infected patients compared to uninfected controls [294]. Similarly, Sadeghi et al. reported reduced levels of Tregs as well as the transcription factors retinoic-acid-receptor-related orphan nuclear receptor γ (RORγt) and forkhead box P3 (FoxP3) in severe COVID-19 patients [287]. In contrast, a study conducted by Tan et al. found similar Treg counts between control and severe COVID-19 patients, which were higher compared to mild patients. This might highlight the negative regulatory role of Tregs in infected patients as their reduction seems to promote viral clearance [295]. Thus, the role of Tregs in COVID-19 remains unclear, which could be due to the timing of sampling from COVID-19 patients or to differences in the classification of patients upon admission. Therefore, further exploration of the regulatory and suppressive functions of these cells following SARS-CoV-2 infection as well as their influence on disease severity and patient survival is critically needed.

Similar to T cells, a reduction in B cells have been reported in COVID-19 patients, thus it has been found to be less pronounced compared to T cells [284,296,297]. Further, recent studies have shown that COVID-19 patients display reduced counts of naïve B cells and increased levels of the exhaustion phenotype, CD21^low^ B cells, which may explain the reduced B cell counts found in COVID-19 patients [284]. Interestingly, SARS-CoV-2 specific antibodies are detectable in the serum of COVID-19 patients, including IgG, IgM, IgA, and IgE [275,284,298]. In line with this finding, Wen et al. reported elevated levels of plasma cells in COVID-19 patients [299]. However, higher antibody titters and early seroconversion were found to be associated with increased severity in COVID-19 [275,298,300–302]. It is not clear why SARS-CoV-2 infected patients develop severe complications and sometimes fail to produce protective immunity against a subsequent infection while their antibody response seems to be unaffected. Thus, further studies are needed to explain this phenomenon.

Further, NK cells are innate lymphoid cells which can directly kill virally-infected cells, have also been found to be reduced in COVID-19 patients [70,299,303,304]. A retrospective study conducted on COVID-19 patients revealed a significant decrease in NK cell counts in severe cases, especially of the cytotoxic NK subset characterized as CD3^−^CD56^dim^CD16^+^ [268]. In addition, several studies have reported the negative influence of SARS-CoV-2 infection on NK cell functions. Serum levels of perforin and Granzyme A (GrA) are reportedly significantly reduced in severe cases of COVID-19 compared to mild cases [268,305]. Thus, this indicates a dysregulated cytotoxic response and impaired ability to inhibit viral replication in severe COVID-19 cases. Although NK cells were found to be significantly reduced in COVID-19 patients, a significant NK cell accumulation was noted in BALF samples taken from the lungs of SARS-CoV-2 infected patients [306]. This could also be the reason for NK presentation of the exhaustion phenotype, as overstimulation occurring during COVID-19 infection could hamper cytotoxic activities of NK cells and induce cellular exhaustion [70]. In addition, the increased secretion of IL-6 following SARS-CoV-2 infection could supress Granzyme B (GrB) production and the expression of NKG2D necessary for eliminating infected cells and eventually hinder the ability of NK cells to clear infected cells [305].

It became apparent early in the pandemic that higher neutrophil to lymphocyte (NLRs) ratios are predictive of disease severity in COVID-19 patients. A higher NLR was also found to be associated with lymphocyte impairment [302,307–310]. Further, elevated levels of serum neutrophil extracellular traps (NETs) have also been detected in SARS-CoV-2 infected patients. Zou et al. reported increased serum levels of several markers commonly used to detect NET remnants in blood including cell-free DNA, myeloperoxidase-DNA (MPO-DNA), and citrullinated histone H3 (Cit-H3) [311]. The same study also reported that patient serum displayed evidence of NETosis when added to control neutrophils [311]. Taken together, these observations indicate the role of neutrophils and their relation to lymphocyte counts in the pathogenesis of COVID-19.

## Cytokine profile in COVID-19

Analysis of plasma from patients with COVID-19 has revealed increases in cytokines, including IL-1β, IL-2, IL-6, IL-7, IL-10, TNFα, monocyte chemoattractant protein (MCP1/CCL2), granulocyte colony stimulating factor (G-CSF), and macrophage inflammatory protein 1α (MIP1α/CCL3). Of this extensive list, recently published studies have zeroed in on several cytokines thought to be key players during SARS-CoV-2 infection. A cohort study by Huang et al. included 41 patients with COVID-19 and revealed that those requiring ICU admission (32%) presented with higher levels of cytokines, including IL-2, IL-7, IL-10, TNFα, G-CSF, CCL2, CCL3, and CXCL10 [7]. Similarly, Zhou et al. examined samples from 191 patients with confirmed COVID-19, of which 54 died, and found that IL-6 levels were higher in non-survivors than survivors [300]. Additionally, a recent retrospective study collected data from 121 patients with COVID-19 and found increased levels of IL-6 (35.2%) and IL-10 (64.4%) on hospital admission [312]. These findings strongly support the involvement of IL-6 and IL-10 in disease prognosis and outcomes during the early stages of COVID-19.

IL-10 is a pleiotropic cytokine mainly produced by macrophages and T cell subsets (Th and Treg), in addition to a wide variety of other immune cells. including DCs, NK and B cells [313]. *In vivo* and *in vitro* studies have shown that IL-10 suppresses the function of pro-inflammatory cytokines, including IL-1α, IL-1β, IL-6, IL-8, IL-12, IL-18, TNFα, G-CSF and M-CSF [313–315]. Moreover, studies on monocyte/macrophage activation found that IL-10 restricts inflammatory responses by inhibiting the expression of MHC II and the co-stimulatory molecules, CD80/CD86, on APCs [316,317]. This is crucial because antigen presentation in adaptive immunity, specifically in relation to Th subsets, is driven by the expression of MHC II molecules on DCs. This finding indicates that the immunosuppression observed in COVID-19 patients is mainly driven by IL-10, hence blocking this cytokine may lead to better outcomes following SARS-CoV-2 infection.

## Discussion

Lymphocytopenia is a common laboratory finding among patients with COVID-19. This is troubling, as lymphocytes are crucial to the adaptive immune response needed to fight infection. Moreover, reported cases of COVID-19 indicate that recovered patients present with increased lymphocyte counts relative to non-recovered patients, who exhibit persistently depressed lymphocyte numbers. This difference clearly indicates that immunosuppression is a pathological phenomenon predictive of complicated patient outcomes. Overall, this review summarizes the major immunological events that occur following SARS-CoV-2 infection, providing an in-depth analysis of key cellular components and cytokines involved in the immunosuppression observed in COVID-19. Collective data indicate that anti-inflammatory cytokines, including IL-10, are likely produced to limit the cytokine storm observed following SARS-CoV-2 infection; however, IL-10 fails to achieve this, as high levels of IL-6 in conjunction with IL-10 are associated with severe cases of COVID-19. Hence, it is possible that, in COVID-19 the induction of IL-10 solely serves to suppress adaptive immunity. Interestingly, through this function, IL-10 may help provide a supportive environment for the development of a cytokine storm, rather than facilitating its elimination. As a pleiotropic cytokine, IL-10 have also been shown to promote inflammatory cytokine production in autoimmune diseases and cancers [318,319]. Interestingly, blocking IL-10 activity have been reported to promote active proliferation and expansion of T cells in cancer patients, thus facilitate the resolution of the immunosuppression observed in these patients [319].

Further, as lymphocyte counts are considered reliable predictors of disease severity and mortality in COVID-19 patients, it is critical for SARS-CoV-2 infected patients to overcome lymphocyte suppression, especially with regard to their CD4 T cell counts. A previous study found that in a SARS-CoV infected mouse model, depletion of CD4 but not CD8 T cells lead to enhanced immune-mediated interstitial pneumonitis and delayed clearance of SARS-CoV from the lung [320]. This finding highlights the critical role of T cells, and especially CD4 T cells, in orchestrating adaptive immune responses following SARS-CoV infection. Further, infection of T cell deficient mice with murine coronavirus revealed that T cells temper early overactive innate responses and limit the cytokine storm observed following coronavirus infection [321]. Consistent with this finding, collective evidence indicates that viral clearance and T cell loss may lead to overactive innate immune responses during viral infections [308]. These observations strongly point to the importance of T cells not only to promote viral clearance, but also in limiting the pathological consequences of the cytokine storm. Further, animal models of chronic infection have been shown to reverse T cell exhaustion following IL-10 blockade [288,289]. Thus, as IL-10 may be responsible for this depression, either by inducing T cell reduction or exhaustion, it is possible that IL-10 blockade may serve as a potential therapeutic approach for COVID-19. It is possibly that by inhibiting IL-10, adaptive immune responses could facilitate the resolution of the cytokine storm through limiting the exaggerated innate immune response, and at the same time reduce the pathological levels of IL-6 produced by innate immune cells. It should be noted that the role of IL-10 in blocking the activity of IL-6 may be impaired in COVID-19 patients; however, further studies are needed to confirm this theory.

## Concluding remarks

Review of currently available data indicate that IL-10 may be the main factor driving the immunosuppression observed following SARS-CoV-2 infection. Furthermore, in the case of COVID-19, the production of counterbalancing anti-inflammatory cytokines, specifically IL-10, may not function to limit the cytokine storm as previously thought, but rather enhance the pro-inflammatory environment in which a cytokine storm can develop. Based on these observations, immunomodulatory approaches for stimulating adaptive immunity, particularly CD4 T cells, through IL-10 blockade, have potential as a therapeutic approach for patients with COVID-19.

## Data Availability

The authors confirm that the data supporting the findings of this study are available within the article.
